# The Effect of Time, Roasting Temperature, and Grind Size on Caffeine and Chlorogenic Acid Concentrations in Cold Brew Coffee

**DOI:** 10.1038/s41598-017-18247-4

**Published:** 2017-12-21

**Authors:** Megan Fuller, Niny Z. Rao

**Affiliations:** Department of Chemistry and Biochemistry, Thomas Jefferson University, East Falls Campus, Philadelphia, PA 19144 USA

## Abstract

The extraction kinetics and equilibrium concentrations of caffeine and 3-chlorogenic acid (3-CGA) in cold brew coffee were investigated by brewing four coffee samples (dark roast/medium grind, dark roast/coarse grind, medium roast/medium grind, medium roast/coarse grind) using cold and hot methods. 3-CGA and caffeine were found at higher concentrations in cold brew coffee made with medium roast coffees, rather than dark roast. The grind size did not impact 3-CGA and caffeine concentrations of cold brew samples significantly, indicating that the rate determining step in extraction for these compounds did not depend on surface area. Caffeine concentrations in cold brew coarse grind samples were substantially higher than their hot brew counterparts. 3-CGA concentrations and pH were comparable between cold and hot brews. This work suggests that the difference in acidity of cold brew coffee is likely not due to 3-CGA or caffeine concentrations considering that most acids in coffee are highly soluble and extract quickly. It was determined that caffeine and 3-CGA concentrations reached equilibrium according to first order kinetics between 6 and 7 hours in all cold brew samples instead of 10 to 24 hours outlined in typical cold brew methods.

## Introduction

In 2015, domestic coffee consumption in the United States reached an estimated 1.4 billion kg/year, making it the second largest coffee market in the world after the European Union^1^. The majority of coffee consumed in the United States is prepared through various hot brewing methods, whereby the hot water solubilizes and extracts numerous organic compounds from the roasted coffee grounds. However, cold brew coffee preparation techniques have grown in popularity, both in at-home and consumer (or ready-to-drink, RTD) markets. Market researcher, StudyLogic, estimates that coffee shop sales of hot coffee fell 3% in 2016, while cold brewed coffee sales were up nearly 80% over the previous year’s record^2^. Roast Magazine reports a 460% increase in retail sales of refrigerated cold brew coffee from 2015 to 2017, generating $38 million in 2017 alone^3^. In an effort to capitalize on this rapidly growing market, Dunkin’ Donuts, Starbucks, and other commercial coffee vendors have invested in RTD cold brew coffee beverages and are suggesting that colder, slower brewing processes alter flavor, aroma, and bioactive compounds^4^. Starbucks markets that cold brew coffee is sweeter, smoother, with a more full-bodied flavor than conventionally brewed coffee^5^. Dunkin’ Donuts claims that, “cold brew is less acidic and naturally sweeter than regular coffee, so it can easily be consumed black”^6^.

Cold brew coffee, not to be confused with iced coffee (which is hot brewed coffee served over ice), is prepared at room temperature (20 to 25 °C or colder) over a longer time period than traditional hot brewing methods, typically steeping times range from 8 to 24 hours^7–10^. Brewing coffee is an extraction process dependent on a multitude of variables such as water volume, water temperature, diameter of the coffee grind particles, the porosity of the coffee grind matrix, the pore network between coffee grind particles, and brewing time. Temperature often significantly influences compounds aqueous solubility, so differences in brewing temperatures may result in significantly different compositions in hot brew and cold brew coffees. Additionally, the longer brewing times of cold brew coffee may affect the final composition of cold brew coffee if the diffusion of the compounds across the grind matrix is a kinetically limiting phenomenon.

An extensive body of literature exists detailing the chemistry of hot brewed coffee, including quantifying the caffeine concentration as a function of hot water brewing method^11–14^. Bioactive chemicals such as chlorogenic acids, caffeine, and other dietary phenolic compounds that include caffeoylquinic acids, dicaffeoylquinic acids, and feruloylquinic acids^15^ are abundant in coffee. These chlorogenic acid compounds convey bitterness to coffee^11^ and are known to be active antioxidants that may cause health benefits in coffee drinkers^16–18^. The presence of these bioactive compounds in the complex chemical composition of coffee extracts have prompted numerous epidemiological studies to ascertain the degree and manner in which coffee confers health risks and/or benefits^19^ to the drinker. Researchers have observed both U-shaped^19–21^ and J-shaped^22^ associations between coffee consumption and risks of cardiovascular diseases. Inverse relationships have been found with coffee consumption and total mortality^23,24^, depression^25^, diabetes mellitus^26,27^ and certain types of cancers^28–30^. Work by Bakuradze, *et al*. showed compounds present in coffee roast products, notably 5-caffeoylquinic acid and caffeic acid demonstrated direct antioxidant activity in HT-29 (human colon) cells^31^. A recent review by Naveed *et al*. further highlighted the therapeutic roles of chlorogenic acids in human health and called for further research in the area^32^. These studies often focus on analysis of green coffee beans, hot brewed coffee consumption, or make no distinction to the brewing method used. Research in hot brew coffees show that both roasting temperature^33,34^ and grind size^35,36^ affect the extraction kinetics and maximum extractable concentration of soluble compounds from coffee grinds, specifically chlorogenic acids^37–40^. Increases in roasting temperatures correlate to a decrease in extractable chlorogenic acid concentrations and to an increase in caffeine concentrations^33^. However, because of the potential chemical differences between hot and cold brew coffee, it is unknown if the new popular drink will convey similar benefits to its hot brew counterpart.

Despite the increasing popularity of cold brew coffee, there is currently very little research published on the chemistry or associated health risks and/or benefits of cold brew coffee. An exhaustive literature search revealed very limited publications analyzing cold brew coffee. In 2014, Kim and Kim reported that the flavor of cold brew coffee may be more appealing to the Korean coffee consumers after 18 hours brewing time^41^. In 2017, Lane *et al*. reported that caffeine concentrations of commercially brewed cold brew coffee was ~207 mg per 12 fl. oz^42^. A third study by Shin in 2017 reported that the polysaccharides isolated from cold brew coffee “may potentially enhance macrophage functions and the intestinal immune system”^43^. To date, these publications represent the majority of published research available on cold brew coffee. Commercial vendors’ claims of lower acidity and other taste and chemical attributes have yet to be verified by unbiased research.

Given cold brew coffee’s significant growth in the coffee market and the potential importance of coffee’s bioactive compounds on human health, this research investigated the role of cold brewing methods on the kinetics and equilibrium conditions of two compounds of interest: caffeine and 3-chlorogenic acid (3-CGA). The pH of all coffees produced in this research was also measured to determine if cold brew coffee does results in a less acidic coffee beverage. This work studied the extraction kinetics of caffeine and 3-chlorogenic for both cold and hot brew methods using single origin Arabica beans grown in the Kona Region of Hawai’i in order to determine the effect water temperature and brewing time on the extraction kinetics and maximum equilibrium concentration of these two bioactive compounds. Brewing methods employed in this work mimicked standard home-brewing conditions to inform what, if any, differences consumers can expect between hot and cold brew coffees.

## Results

### Kinetics of cold brew coffee extraction

Four coffee samples were used in this study. See Table 1 for grind size distribution and roasting temperature characteristics for each of the samples.

**Table 1:** Summary of grind size distribution by percent mass (100.0 g of grinds used in each analysis) and roasting temperature, as reported by the coffee vendor.

Sample Name (Roast - Grind)	Grind Size (% by mass)	Roasting Temperature
Medium - Medium	3350 µm - 5.7% 841 µm - 26.2%400 µm - 53.3%149 µm - 14.8%	215 –217 °C
Medium - Coarse	3350 µm - 0% 841 µm - 70.6%400 µm - 23.1% 149 µm - 6.3%	215 –217 °C
Dark - Medium	3350 µm - 5.2% 841 µm -38.1%400 µm - 45.4% 149 µm - 11.3%	223 –225 °C
Dark - Coarse	3350 µm - 0% 841 µm - 77.8% 400 µm - 17.5% 149 µm - 4.3%	223 –225 °C

The grain size distributions of the four samples show that the “medium” grind coffees had wider particle distributions, both containing about 5% of particles, by mass, that are larger than 3350 µm. The “coarse” grind coffees showed no 3350 µm portion, and have a narrower particle distribution with more than 70% of particles, by mass, being retained on the 841 µm sieve. Coffee beans are naturally porous. The pore space within each grain of coffee is considered the intragranular pores. The space between grains of coffee is referred to as the intergranular pores. Earlier studies found that particle distribution was vitally important to coffee extraction, affecting both the diffusion of compounds through intragranular pores within grinds, as well as the fluid flow between the grinds (through the intergranular pore network)^14,44,45^. The importance of both intragranular and intergranular pore space will be discussed further with respect to diffusion limiting processes of compound extraction kinetics.

#### 3-CGA

**Figure 1:**
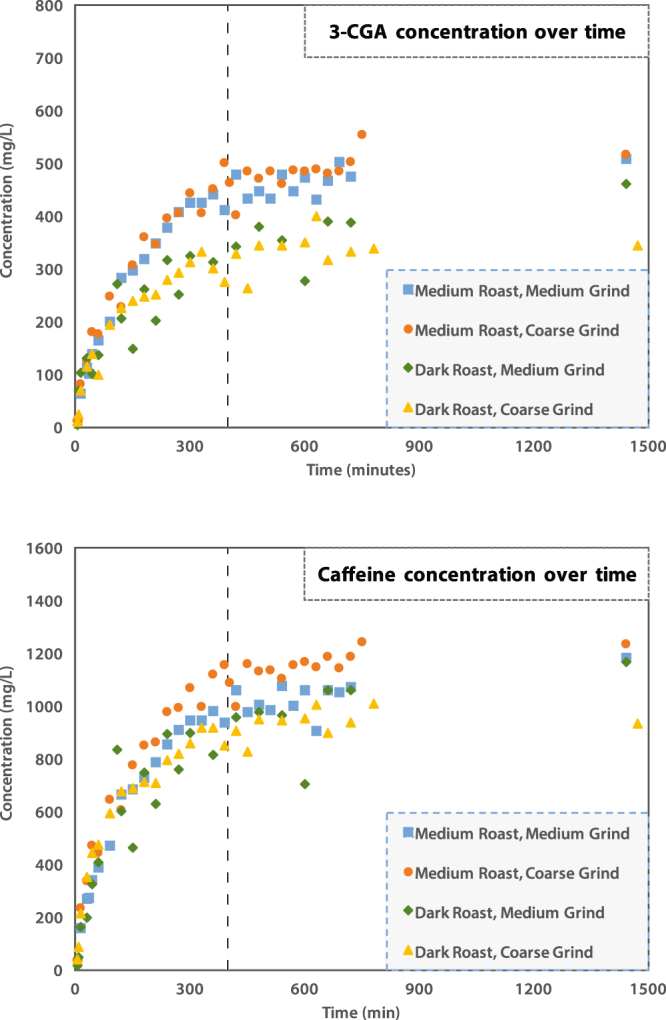
Concentration of 3-CGA (top) and caffeine (bottom) over time for (■) medium roast - medium grind; (●) medium roast - coarse grind; (◆) dark roast – medium grind, (▲) dark roast - coarse grind. The vertical line at 400 minutes represents the establishment of steady-state concentration for both 3-CGA and caffeine extractions.

**Figure 2:**
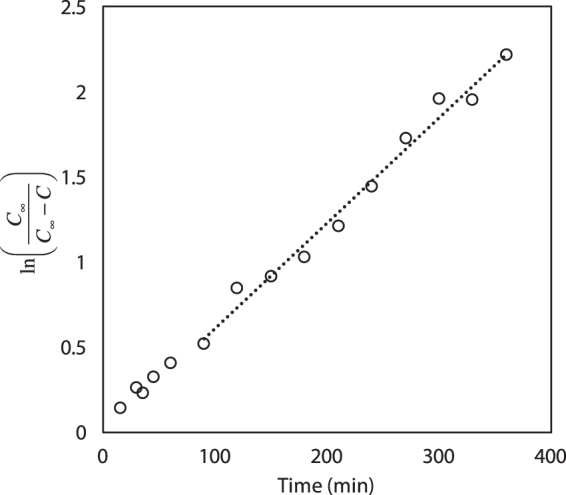
First-order plot for the extraction of 3-CGA from medium grind - medium roast coffee particles at 23.5 °C. R^2^ = 0.983.

The compound, 3-CGA is freely soluble in water at room temperature^46^. Initial 3-CGA concentration increased rapidly over the first 180 minutes and slowed until reaching equilibrium at approximately 400 minutes for all coffee roasts and grinds (see Fig. 1). Moroney *et al*.^45^ attributed the initial fast extraction of soluble coffee compounds to the extraction of compounds from the surface and near-surface volume of the solid coffee grind matrix. The slower, longer time-scale extraction of additional CGA concentration, post 180 minutes, is likely due to the mass transfer of the compound through intra-grain pores into intergrain pores, and ultimately into the bulk liquid phase. The data collected in this current work follows the Spiro and Selwood^36^ model well and suggests that cold brew processes of 3-CGA extraction are governed by first-order kinetics. A sample kinetic plot of ln (C_∞_/[C_∞_− C]) for 3-CGA versus time is shown in Fig. 2 where C_∞_ is the equilibrium concentration of 3-CGA and C is the concentration of 3-CGA at time *t*. Several sources^7–10^ providing brewing instructions for cold brew coffee recommend prolonged brewing times upwards of 12 to 24 hours. Any water/grind interaction longer than 400 minutes (6.7 hours) did not result in additional significant extraction of 3-CGA. The mean concentrations of 3-CGA at 400 and 1400 minutes were within one standard deviation of each other (see Table 1). These data suggest 3-CGA concentrations are influenced by roasting temperature, but not grind size. Blumberg *et al*.^11^ determined that increased roasting temperatures resulted in degradation of chlorogenic acid precursors and lower extractable total chlorogenic acid concentrations. The same study also observed that chlorogenic acids extracted quickly from coffee grinds, while 4-vinylcatechol oligomers showed strong retention to the coffee grinds^11^. The longer steeping times associated with cold brew coffee may result in increased extraction of these catechol oligomers, which are characterized by harsh bitter-tasting properties. Over-brewing cold brew coffee may result in unpalatable extracts due to these and other relatively slow-extracting compounds.

#### Caffeine

Caffeine, unlike 3-CGA, has a limited solubility at room temperature of 16 mg/mL^46^. However, all four coffees analyzed showed comparable extraction kinetics to those of 3-CGA. In all coffees sampled, fast initial extraction was seen over the first 180 minutes, with a slower rate of extraction after 180 minutes. Similar to 3-CGA, caffeine also reaches nearly steady-state concentrations after 400 minutes (see Fig. 1). As with 3-CGA, all samples followed a first-order kinetic model. Spiro and Selwood^36^ offered a thorough mathematical model for the kinetics of caffeine extraction at room temperature, and found that the diffusion of caffeine through the intragranular pore space to be the rate limiting step in the extraction process. This analysis concluded that extraction times greater than 400 minutes do little to increase the caffeine concentration of the resulting coffee. Moreover, caffeine concentrations do not demonstrate the same sensitivity to roasting temperatures as 3-CGA, and all coffee roasts and grinds were found to have comparable caffeine concentrations at equilibrium, with the exception of the dark roast - coarse grind coffee. The relatively high standard deviations are suspected to be caused by the heterogeneous grind size distributions from the commercially sourced beans. As the packaging was handled, settling of finer particles may have caused inter-sample variability.

#### pH

Work by Andueza *et al*.^47^ and Gloess *et al*.^48^ both report there is no correlation between pH and perceived acidity in the flavor of coffees. However, commercial coffee vendors continue to relate acidity to coffee taste when marketing coffee to consumers. The pH of coffee studied in this work ranged from 5.40 to 5.63. Moon *et al*.^15^ observed a correlation between total CGA concentrations and pH of coffee extracts. However, data collected in this work did not provide enough evidence to support the claim by Moon *et al*.^15^ that coffee samples containing high concentration of 3-CGA tend to have high acidity or low pH.

### Comparison of hot brew and cold brew coffee

There is a common marketing message that cold brew coffee is fundamentally different than hot brew coffee. This may be attributed to acidity and/or caffeine concentration^49,50^. This work compared the same water-to-coffee ratio using cold brew and hot brew extraction processes to identify any differences between the two methods with respect to 3-CGA and caffeine concentrations. In the coffee extraction process, Moroney *et al*.^35^ described two different extraction mechanisms that function on different timescales. The fast extraction from the surface and near-surface matrix happens much more rapidly than the diffusion of compounds through the intragranular pore network to the grain surface. Because the time periods for hot brew and cold brew are drastically different, 6 minutes vs. 1440 minutes respectively, the intragranular diffusion may limit the extractable concentration of soluble coffee compounds in the hot brew, as compared to the cold brew.

#### 3-CGA

**Figure 3:**
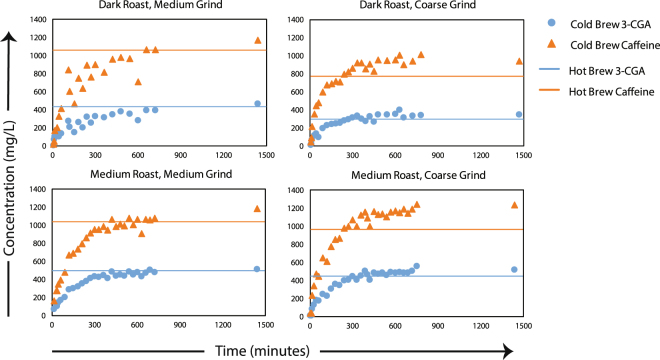
Caffeine (▲) and 3-CGA (●) concentration as a function of time for each of the four coffee samples. Horizontal lines represent each coffee type’s hot water concentration for caffeine and 3-CGA. Error bars represent the range for each measurement.

In Fig. 3, the cold brew extraction of caffeine and 3-CGA are shown for each of the four coffee samples, with the hot brew concentrations indicated by horizontal lines. Table 2 shows the equilibrium concentrations of 3-CGA for the hot and cold brew coffees along with the pH. In both hot and cold brew extractions, all samples show comparable average 3-CGA concentrations and pH, regardless of water temperature. The CGA molecule is not seen to be limited by the intragranular pore diffusion processes, as observed with caffeine extraction. CGA is freely soluble in water, and this facilitates its extraction at both low and high temperatures. While grain size did not impact the magnitude of 3-CGA concentrations, roasting temperature of the beans did show a noticeable effect in both cold and hot experiments. In both hot and cold brew extractions, CGA was found in higher concentrations in medium roasts than in darks roasts, supporting the work of Trugo and Macrae^37^ that suggests that higher roasting temperatures decomposes CGA and results in lower extraction concentrations.

**Table 2:** Concentration of 3-CGA and caffeine and pH of each cold brew coffee sample after 400 minutes and 1440 minutes of brewing time (Mean ± 95% Confidence Interval, *n* = 6).

Coffee Sample (Roast - Grind)	400 min			1440 min		
	3-CGA Concentration (mg/L)	Caffeine Concentration (mg/L)	pH	3-CGA Concentration (mg/L)	Caffeine Concentration (mg/L)	pH
Medium - Medium	480 ± 60	1060 ± 60	5.61 ± 0.01	510 ± 20	1180 ± 90	5.54 ± 0.02
Medium - Coarse	490 ± 30	1130 ± 50	5.47 ± 0.01	520 ± 40	1230 ± 60	5.40 ± 0.01
Dark - Medium	380 ± 10	970 ± 60	5.63 ± 0.01	390 ± 10	1080 ± 70	5.53 ± 0.01
Dark - Coarse	330 ± 50	930 ± 40	5.51 ± 0.02	360 ± 20	990 ± 30	5.41 ± 0.02

### Caffeine

Coarse grain samples, both medium and dark roast, showed a considerable deviation in caffeine concentrations between hot and cold brew extractions (Table 3). In both samples, the cold brew coffee was found to have the higher concentration of caffeine. Medium grain samples also showed higher concentrations of caffeine in cold brew extraction, however, the difference was not statistically significant. This result suggests that the medium grind blends, in both hot and cold extraction, experienced nearly complete extraction during their respective steeping times. The hot brew extraction saturated the intra- and intergranular pores and facilitated fast diffusion (6-minute steeping times) of caffeine across the solid matrix to generate a bulk liquid phase with nearly the same concentration of caffeine as the cold brew coffee generated in 400 minutes. Coarse grain samples, with their higher relative proportion of particles in the 841 µm range, did not reach similar steady-state concentrations in both hot and cold brews. The faster, hot water extraction was diffusion limited, and likely did not allow the full extraction of caffeine across the larger radius particles. The longer brewing times for the cold brew samples resulted in greater caffeine extraction, allowing time for completion of the rate-limiting mass transfer step in the extraction process.

**Table 3:** Comparison of equilibrium 3-CGA and caffeine concentrations (after 1440 min) extracted using cold and hot brew method along with pH (mean ± 95% Confidence Interval, *n* = 6).

Coffee Sample (Roast - Grind)	3-CGA Concentration (mg/L)		Caffeine Concentration (mg/L)		pH	
	Cold Brew Method	Hot Brew Method	Cold Brew Method	Hot Brew Method	Cold Brew Method	Hot Brew Method
Medium - Medium	510 ± 20	510 ± 30	1180 ± 90	1040 ± 70	5.54 ± 0.02	5.41 ± 0.02
Medium - Coarse	520 ± 40	460 ± 40	1230 ± 60	970 ± 70	5.40 ± 0.01	5.35 ± 0.03
Dark - Medium	390 ± 10	430 ± 30	1080 ± 70	1060 ± 70	5.53 ± 0.01	5.61 ± 0.02
Dark - Coarse	360 ± 20	340 ± 10	990 ± 30	840 ± 10	5.41 ± 0.02	5.48 ± 0.02

### Role of Grind Size and Roasting Temp in Cold Brew Coffee

Further analysis of the data indicates that the observed CGA and caffeine concentration differences between medium roast and dark roast are, in general, substantial. Both CGA and caffeine showed higher concentration in medium roast samples. Our data is in support of the works of Trugo, *et al*.^37^ and Hečimović, *et al*.^51^, both suggest that higher roasting temperatures decrease the concentration of CGA and caffeine. The only exception is the observed difference in concentration of caffeine when comparing medium roast – medium grind and dark roast – medium grind samples. Although the medium roast samples showed higher concentration of caffeine than dark roast samples, the observed difference in concentration is insignificant due to large variations in the measurements.

## Discussion and Conclusional Remarks

This work establishes that brewing times near 400 minutes are adequate to extract the majority of available caffeine and 3-CGA in medium and dark roast beans prepared at medium and coarse grinds. Moreover, coarse grain samples, both medium and dark roast, showed a substantial increase in caffeine concentrations than their hot brew counterparts. No significant differences were seen in CGA concentrations between cold and hot brews. Furthermore, the pH between cold and hot brews were comparable. This work suggests that any claims made by coffee vendors about the difference in acidity or taste of cold brew coffee is not due to variations in 3-CGA concentrations. The results of this study validate earlier models proposed by by Moroney *et al*.^35,45^ and Spiro and Seldwood^52^ and extend their findings into cold brew extraction over long time periods.

**Figure 4:**
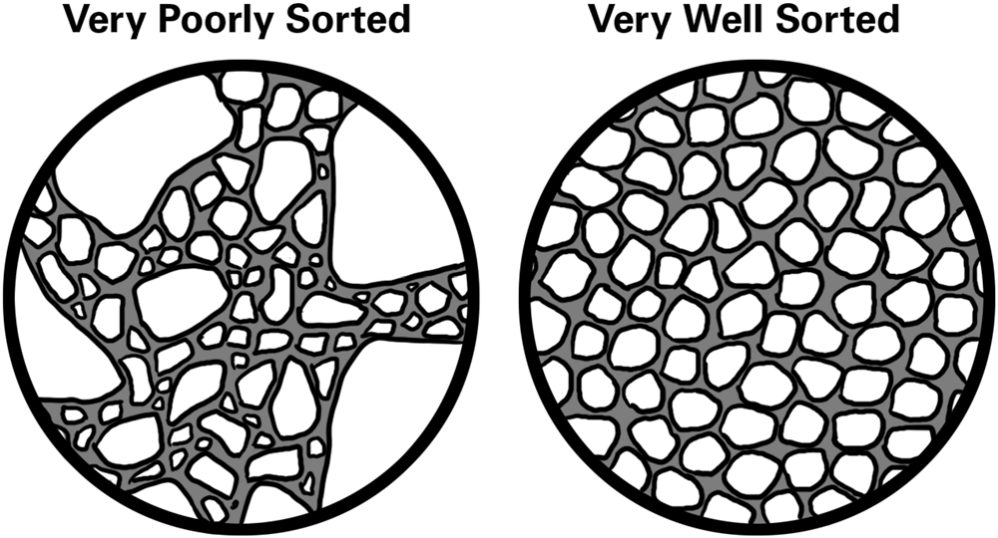
The graphical image of sorting effects on pore connectivity in coffee matrix beds. Grain size distribution influences both the intragranular diffusion process, as larger grains have greater diffusion distances, and the intergranular pore network, as poorly sorted grain beds have more tortuous pore networks.

Furthermore, our analysis indicates that the grind size does not have significant impact the observed equilibrium concentrations for both CGA and caffeine. When comparing samples with the same roasting temperatures, the observed differences in concentrations are largely within one standard deviation from another. Mathematical modeling of coffee extraction proposed by Moroney, *et al*.^35,45^ suggested that the diffusion of coffee from the intragranular pores to the intergranular pores is the rate limiting process. Thus, it takes longer for the extraction process to reach equilibrium as the grind size increases. However, in the cold brew process, the extraction time frame is on the order of hours instead of seconds. Such long extraction time scales allow for the slow diffusion from intragranular to intergranular pores, so this is not a rate determining process in cold brew methodologies. We have noted that grind size was not well controlled in this study, as this work used commercially available coffee without any size separation prior to extraction. Future work will differentiate coffee grinds by particle size to further quantify the role of grind size in cold brew coffee. Figure 4 shows a graphical representation of a poorly sorted and a well sorted coffee grind. Grind size and grind sorting are both important parameters controlling inter- and intragranular diffusion.

## Materials and Methods

### Materials

Coffee from the Kona region of Hawai’i was sourced from Kona Joe Coffee, LLC (*Kealakekua, HI*). This single origin Arabica (*Kona Typica*) coffee was obtained roasted and ground from Kona Joe Coffee following their standard preparation processes. Four coffee types were used in this study. Two roasting temperatures, medium and dark, prepared at two grind sizes, medium and coarse were selected for this work. Both roasting and grinding were done by Kona Joe Coffee. Medium roast beans were roasted at 215 °C to 217 °C, Dark roast beans were roasted at 223 °C to 225 °C. The beans were ground using a Mahlkönig DK-15 industrial grinder.

Standard stock solutions of 400 mg/L caffeine and 3-CGA were made daily and diluted to establish calibration curves for coffee analysis. Both purchased from Sigma Aldrich (*Milwaukee, WI*). HPLC grade methanol was obtained from Fisher Scientific (*Nazareth, PA*). Phosphoric acid (85% wt.) was obtained from Sigma Aldrich (*Milwaukee, W*I) and diluted to 2.0 mM concentration using DI water. Filtered municipal tap water used in this study. Analysis of this water, completed by Penn State University’s Agricultural Analytical Services Laboratory found the water to have a total hardness of 174 mg/L and a pH of 7.5.

### Methods

#### Particle size distribution

The particle size distributions of each grind were determined according to ASTM C136/C136M-14 Standard Test Method for Sieve Analysis for Fine and Coarse Aggregates procedure. Samples of 100.0 g of coffee grinds were added to a sieve stack including sieve sizes #20 (0.841 mm mesh opening), #40 (0.420 mm mesh opening), #100 mesh (0.149 mm mesh opening), and pan, to generate grain size distributions for each coffee used in this study.

#### Cold brew experiments

The cold brewing process was carried out at room temperature (ranging from 21 °C to 25 °C over the experimental period) adapted from a home-brewing recipe suggested by The New York Times’ *Cooking* website^9^. A sample of 35.0 g of coffee was placed in 350 mL of carbon-filtered municipal water. The coffee was contained in T-Sac™ tea filter bag (size 4) and placed in a 32-ounce Mason jar fitted with a screw-top lid. The filter bag was used to reduce grind loss during sampling and ensure grinds remained submerged during steeping. The coffee/water mixture was sampled every 15 minutes for the first hour, then every 30 minutes until hour 7, and then once an hour until hour 12, a final sample was taken at 24 hours. Samples collected after the first hour were diluted (1:4) with DI water and filtered using HT Tuffryn (*Pall*) 25 mm diameter, 0.2 μm pore size membranes. Fresh water was added to replace the volume sampled to maintain constant volume. This introduced a small dilution effect in the resulting solution. Additionally, even with the closed system, there was inevitably evaporation over the 24 hour testing period. Coffee received from Kona Joe Coffee was not processed in any way prior to use, to best match home-brewing conditions. Data presented are an average of triplicate experiments analyzed in duplicates (*n* = 6).

#### Hot brew experiments

Hot brew extraction was conducted using the same coffee to water ratio as was used in the cold brew method. The water was heated to 98 °C and added to coffee grounds in a traditional French press carafe. The water and grounds were allowed to sit for 6 minutes before the filter was depressed and the coffee decanted. Since additional experiments showed that longer mixing times did not result in additional caffeine or 3-CGA extraction, 6-minute extraction times were used for all hot brew experiments. Two samples were taken from each hot brew and each experiment was performed in triplicate (*n* = 6).

#### Caffeine and 3-CGA measurement

Caffeine and 3-CGA were measured in both standard solutions and coffee extracts using an adapted methodology reported in GL Sciences Technical Note No. 67^53^. An Agilent 1200 Series high performance liquid chromatography system (HPLC) was fitted with a Supelco 5 µm column (15 cm × 4.6 cm) (*Supleco, Bellefonte, PA*) run at 40.0 °C with a mobile phase mixture of 75% mobile phase A and 25% mobile phase B (A: 95% 2.0 mM phosphoric acid and 5% methanol; B: 95% methanol and 5% 2.0 mM phosphoric acid). The flow rate was 1.0 mL/min with an injection volume of 10.0 µL. Caffeine and 3-CGA were detected using a diode array detector at 280 nm and 325 nm respectively.

#### pH measurements

The pH of each brewed coffee sample was measured with a Mettler Toledo FiveEasy^TM^ F20 benchtop pH/mV meter.

#### Statistical Analysis

Two-tailed t-test and ANOVA were employed for determination of similarities in equilibrium concentrations of 3-CGA and caffeine with consideration of the roast, grind size, and brewing method. The output of the statistical analysis is included in the supporting information.

#### Data Availability

All data generated or analyzed during this study are included in this published article (and its Supplementary Information files).

## Electronic supplementary material

Supporting Information
