# Supplementary material for: The Effect of Time, Roasting Temperature, and Grind Size on Caffeine and Chlorogenic Acid Concentrations in Cold Brew Coffee
Source: Sci Rep. 2017 Dec 21;7:17979. doi: 10.1038/s41598-017-18247-4 (PMC5740146; doi:10.1038/s41598-017-18247-4)
Supplement: Supplementary file 1 — Supporting Information [file 41598_2017_18247_MOESM1_ESM.pdf]

Supporting Information:

The Effect of Time, Roasting Temperature, and Grind Size on Caffeine and Chlorogenic Acid Concentrations in Cold Brew Coffee

Megan Fuller[1,*], Niny Z. Rao[1]

[1]Department of Chemistry and Biochemistry, Thomas Jefferson University, East Falls Campus, Philadelphia, PA 19144, USA

* Corresponding Author
Megan Fuller, Ph.D.
Thomas Jefferson University
East Falls Campus
Philadelphia, PA 19144
Telephone: (215) 951-6870
E-mail: fullerm@philau.edu

Table S1. One way ANOVA for the comparison 3-CGA concentrations in 4 coffee samples using the cold brew method.

Analysis of Variance (One-Way)

SUMMARY

Groups	*Count*	*Sum*	*Average*	*Variance*
Medium Roast, Medium Grind	6	3020.47	503.4116667	581.2997767
Medium Roast, Coarse Grind	6	3220.13	536.6883333	689.2994967
Dark Roast, Medium Grind	6	2372.27	395.3783333	253.1773367
Dark Roast, Coarse Grind	6	2113.48	352.2466667	353.8139467

ANOVA

Source of Variation	*SS*	*df*	*MS*	*F*	*P-value*	*F crit*
Between Groups	137215.4701	3	45738.49003	97.44081821	4.16351E-12	3.098391212
Within Groups	9387.952783	20	469.3976392			
Total	146603.4229	23				

Table S2. One way ANOVA for the comparison caffeine concentrations in 4 coffee samples using the cold brew method.

Analysis of Variance (One-Way)

SUMMARY

Groups	*Count*	*Sum*	*Average*	*Variance*
Medium Roast, Medium Grind	6	6967.05	1161.175	4624.00043
Medium Roast, Coarse Grind	6	7843.93	1307.321667	7883.708617
Dark Roast, Medium Grind	6	6643.92	1107.32	12028.74788
Dark Roast, Coarse Grind	6	6082.73	1013.788333	545.1878567

ANOVA

Source of Variation	*SS*	*df*	*MS*	*F*	*P-value*	*F crit*
Between Groups	271339.0437	3	90446.34792	14.42430888	3.10959E-05	3.098391212
Within Groups	125408.2239	20	6270.411196			
Total	396747.2677	23				

Table S3. Two-tailed t-tests for the comparison of 3-CGA concentrations between cold and hot brewing methods.

Medium Roast, Medium Grind

	CGA Cold	*CGA Hot*
Mean	509	512
Variance	524.41	1764
Observations	6	6
Hypothesized Mean Difference	0	
df	7.731445759	
t Stat	-0.153613704	
P(T<=t) two-tail	0.882249032	
t Critical two-tail	2.364624252	

Medium Roast, Coarse Grind

	CGA Cold	*CGA Hot*
Mean	518	455
Variance	2926.81	1900.96
Observations	6	6
Hypothesized Mean Difference	0	
df	9.567988577	
t Stat	2.220971005	
P(T<=t) two-tail	0.053479228	
t Critical two-tail	2.262157163	

Dark Roast, Medium Grind

	CGA Cold	*CGA Hot*
Mean	388	433
Variance	259.21	1391.29
Observations	6	6
Hypothesized Mean Difference	0	
df	6.800590635	
t Stat	-2.713191043	
P(T<=t) two-tail	0.034957519	
t Critical two-tail	2.446911851	

Dark Roast, Coarse Grind

	CGA Cold	*CGA Hot*
Mean	361	343
Variance	761.76	106.09
Observations	6	6
Hypothesized Mean Difference	0	
df	6.36619708	
t Stat	1.496669114	
P(T<=t) two-tail	0.185118934	
t Critical two-tail	2.446911851	

Table S4. Two-tailed t-tests for the comparison of 3-CGA concentrations between cold and hot brewing methods.

Medium Roast, Medium Grind

	Caff Cold	*Caff Hot*
Mean	1183	1040
Variance	15376	6889
Observations	6	6
Hypothesized Mean Difference	0	
df	8.731343422	
t Stat	2.347471417	
P(T<=t) two-tail	0.046865881	
t Critical two-tail	2.306004135	

Medium Roast, Coarse Grind

	Caff Cold	*Caff Hot*
Mean	1233	967
Variance	5791.21	6544.81
Observations	6	6
Hypothesized Mean Difference	0	
df	9.962819612	
t Stat	5.86637376	
P(T<=t) two-tail	0.000238839	
t Critical two-tail	2.262157163	

Dark Roast, Medium Grind

	Caff Cold	*Caff Hot*
Mean	1079	1058
Variance	7191.04	7621.29
Observations	6	6
Hypothesized Mean Difference	0	
df	9.991569977	
t Stat	0.422652294	
P(T<=t) two-tail	0.682467763	
t Critical two-tail	2.262157163	

Dark Roast, Coarse Grind

	Caff Cold	*Caff Hot*
Mean	985	840
Variance	1156	116.64
Observations	6	6
Hypothesized Mean Difference	0	
df	5.998827734	
t Stat	9.956136513	
P(T<=t) two-tail	0.000174589	
t Critical two-tail	2.570581836	

Table S5. Two-tailed t-tests for the comparison of 3-CGA and caffeine concentrations between medium roast and dark roast samples using cold brew method.

CGA

	Medium Roast, Medium Grind	*Dark Roast, Medium Grind*
Mean	509	388
Variance	524.41	259.21
Observations	6	6
Hypothesized Mean Difference	0	
df	8.972356378	
t Stat	10.58786121	
P(T<=t) two-tail	5.53245E-06	
t Critical two-tail	2.306004135	

	Medium Roast, Coarse Grind	*Dark Roast, Coarse Grind*
Mean	518	361
Variance	2926.81	761.76
Observations	6	6
Hypothesized Mean Difference	0	
df	7.437575014	
t Stat	6.332078099	
P(T<=t) two-tail	0.0003919	
t Critical two-tail	2.364624252	

Caffeine

	Medium Roast,	*Dark Roast,*
Mean	1183	1079
Variance	15376	7191.04
Observations	6	6
Hypothesized Mean Difference	0	
df	8.837451764	
t Stat	1.695788419	
P(T<=t) two-tail	0.128368349	
t Critical two-tail	2.306004135	

	Medium Roast,	*Dark Roast,*
Mean	1233	985
Variance	5791.21	1156
Observations	6	6
Hypothesized Mean Difference	0	
df	6.919639995	
t Stat	7.288230387	
P(T<=t) two-tail	0.00033994	
t Critical two-tail	2.446911851	

Table S6. Two-tailed t-tests for the comparison of 3-CGA and caffeine concentrations between medium grind and coarse grind samples using cold brew method.

CGA

	Medium Roast, Medium Grind	*Medium Roast, Coarse Grind*
Mean	509	518
Variance	259081	2926.81
Observations	6	6
Hypothesized Mean Difference	0	
df	5.112954502	
t Stat	-0.043068626	
P(T<=t) two-tail	0.967313848	
t Critical two-tail	2.570581836	

	Dark Roast, Medium Grind	*Dark Roast, Coarse Grind*
Mean	388	361
Variance	259.21	761.76
Observations	6	6
Hypothesized Mean Difference	0	
df	8.049660717	
t Stat	2.069821524	
P(T<=t) two-tail	0.072249115	
t Critical two-tail	2.306004135	

Caffeine

	Medium Roast, Medium Grind	*Medium Roast, Coarse Grind*
Mean	1183	1233
Variance	15376	5791.21
Observations	6	6
Hypothesized Mean Difference	0	
df	8.298481754	
t Stat	-0.841809496	
P(T<=t) two-tail	0.424342625	
t Critical two-tail	2.306004135	

	Dark Roast, Medium Grind	*Dark Roast, Coarse Grind*
Mean	1079	985
Variance	7191.04	1156
Observations	6	6
Hypothesized Mean Difference	0	
df	6.567059545	
t Stat	2.520212907	
P(T<=t) two-tail	0.045274073	
t Critical two-tail	2.446911851	
